# The Protective and Restorative Effects of Growth Hormone and Insulin-Like Growth Factor-1 on Methadone-Induced Toxicity In Vitro

**DOI:** 10.3390/ijms19113627

**Published:** 2018-11-17

**Authors:** Erik Nylander, Sofia Zelleroth, Fred Nyberg, Alfhild Grönbladh, Mathias Hallberg

**Affiliations:** The Beijer Laboratory, Department of Pharmaceutical Biosciences, Division of Biological Research on Drug Dependence, Uppsala University, Uppsala, SE-751 24, Sweden; sofia.zelleroth@farmbio.uu.se (S.Z.); fred.nyberg@farmbio.uu.se (F.N.); alfhild.gronbladh@farmbio.uu.se (A.G.); mathias.hallberg@farmbio.uu.se (M.H.)

**Keywords:** growth hormone, insulin-like growth factor-1, neuroprotection, neurorecovery, cognition, primary cell cultures, methadone, opioids

## Abstract

Evidence to date suggests that opioids such as methadone may be associated with cognitive impairment. Growth hormone (GH) and insulin-like growth factor-1 (IGF-1) are suggested to be neuroprotective and procognitive in the brain and may therefore counteract these effects. This study aims to explore the protective and restorative effects of GH and IGF-1 in methadone-treated cell cultures. Primary cortical cell cultures were harvested from rat fetuses and grown for seven days in vitro. To examine the protective effects, methadone was co-treated with or without GH or IGF-1 for three consecutive days. To examine the restorative effects, methadone was added for the first 24 h, washed, and later treated with GH or IGF-1 for 48 h. At the end of each experiment, mitochondrial function and membrane integrity were evaluated. The results revealed that GH had protective effects in the membrane integrity assay and that both GH and IGF-1 effectively recovered mitochondrial function and membrane integrity in cells pretreated with methadone. The overall conclusion of the present study is that GH, but not IGF-1, protects primary cortical cells against methadone-induced toxicity, and that both GH and IGF-1 have a restorative effect on cells pretreated with methadone.

## 1. Introduction

Growth hormone (GH) is secreted from the anterior pituitary gland and regulates many physiological functions such as growth, bone metabolism, mood, hunger, and sleep [1]. In addition to these effects, GH is also known for its procognitive and neuroprotective actions in the brain [2]. The procognitive effects of GH have been demonstrated in various studies. For instance, GH improves spatial memory in rodents [3,4,5,6] and long-term memory in zebrafish [7], and promotes neurogenesis in mammals [8,9]. Similar effects are seen in humans: GH treatment has been shown to improve cognitive function in patients suffering from GH deficiency [10,11] and traumatic brain injury [12,13], and in a patient on long-term treatment with opioids [14]. The neuroprotective aspect of the hormone has also been demonstrated in several studies [15,16,17], and we have previously reported that GH stabilizes membrane integrity and improves mitochondrial function in primary hippocampal and cortical cell cultures exposed to opioids [18,19]. Both the procognitive and neuroprotective properties are further recognized by the fact that GH is able to cross the blood–brain barrier [20] and that GH receptors are expressed in various brain areas associated with cognition, such as the hippocampus and cortex [21]. 

Many of these effects may be mediated by insulin-like growth factor-1 (IGF-1), which is known to promote many of GH’s peripheral effects [22]. Similar to GH, several studies have demonstrated that IGF-1 improves cognition and acts as a neuroprotectant in the central nervous system (CNS) [23]. Such studies include improved spatial memory in mice [24], neuroprotective and anti-apoptotic effects in cell cultures [25,26], and involvement in neuronal repair [27], as well as studies highlighting the effects of IGF-1 on neurogenesis [9]. Moreover, IGF-1 is essential for normal brain development, as mice with induced disruption of the *IGF-1* gene displayed reduced neuronal development, primarily related to reduced axon growth and myelination [28]. Previous studies also demonstrated that low levels of circulating IGF-1 may be associated with a decline in cognitive function, as seen in the elderly population [29,30]. As both GH and IGF-1 display promising effects with respect to cognition and neuroprotection, it is possible that these hormones can be used as viable treatments for cognitive impairments in the CNS.Accumulating evidence suggest that opioids such as methadone may induce cognitive disorders. Methadone is prescribed as a treatment for heavy pain and is, in many countries, part of the opioid maintenance program for the treatment of heroin addiction. However, several studies indicate that long-term use of methadone reduces cognitive function [14,31,32,33,34,35,36]. Impaired cognitive function was also seen in children born from mothers who participated in an opioid maintenance program [37]. The negative effects caused by methadone, or opioids in general, are associated with increased neuronal cell death [18,19,38,39], reduced neurogenesis [40], and volumetric changes in various brain areas associated with cognition [41]. There is also evidence that opioids may decrease endogenous levels of GH and IGF-1 [14] and reduce the volume of the hypothalamus [41], which may suggest a relationship between opioid use and the somatotrophic axis. 

The negative effects caused by opioids are worrying, and considering the increasing problems associated with them, which include their increased use in young adults [42], frequency of use, mortality, and nonprescribed use [43], these problems need to be further addressed. The promising procognitive effects seen with GH and IGF-1 treatment, as well as the neuroprotective properties, suggest that GH and IGF-1 may be effective pharmacological interventions for opioid-induced cognitive impairment. The present study therefore aims to explore the protective and restorative effects of recombinant human GH (rhGH) and IGF-1 in methadone-treated primary cortical cell cultures. 

## 2. Results

### 2.1. High Concentrations of rhGH, but Not IGF-1, Stabilize Membrane Integrity in Untreated Cells

**Figure 1:**
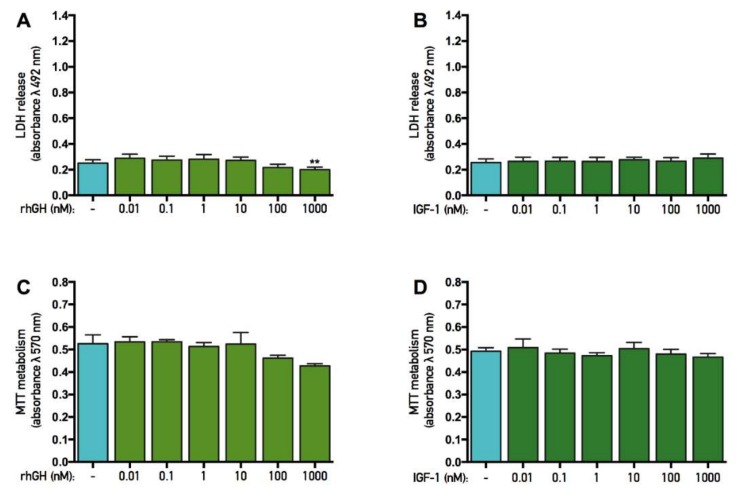
Effects of recombinant human growth hormone and insulin-like growth factor-1 on untreated cells. Mitochondrial function was assessed by tetrazolium bromide (MTT) assay and membrane integrity was assessed by lactate dehydrogenase (LDH) assay. Recombinant growth hormone (rhGH) and insulin-like growth factor-1 (IGF-1) were added for three consecutive days before analysis. (**A**) Effects of rhGH on membrane integrity. A significant decrease in LDH was observed using 1000 nM rhGH compared with control. (**B**) Effects of IGF-1 on membrane integrity. No significant effects were observed when cells were treated with IGF-1. (**C**) Effects of rhGH on mitochondrial function. No significant effects were observed when cells were treated with rhGH. (**D**) Effects of IGF-1 on mitochondrial function. No significant effects were observed when cells were treated with IGF-1. Statistics were calculated using two-way analysis of variance (ANOVA) and given a significant overall effect (*p* < 0.05), the data were further analyzed using Dunnet’s *post hoc* test. All data are presented as mean ± SEM in triplicate from five individual cultures. ** *p* < 0.01.

Both rhGH and IGF-1 were added to untreated primary cortical cells in various concentrations ranging from 0.01 to 1000 nM for three consecutive days. At the end of the experiment, the membrane integrity, as assessed by lactate dehydrogenase (LDH) assay, was evaluated. There was an overall effect of rhGH treatment in untreated cells (ANOVA, *p* < 0.0001), and further *post hoc* analysis revealed that 1000 nM rhGH significantly decreased LDH release by 3% compared with untreated control cells. A numerical, but not significant (*p* = 0.0877), 2% decrease in LDH was observed using 100 nM rhGH (Figure 1A). However, there was no overall effect of IGF-1 treatment in untreated cells (as indicated by ANOVA, *p* = 0.1022) and no further *post hoc* analysis was carried out (Figure 1B). The results from this experiment indicate that high concentrations of rhGH have a general membrane-stabilizing effect, while IGF-1 treatment has no effect on normal untreated cells.

### 2.2. rhGH and IGF-1 Treatment Do Not Alter Mitochondrial Function in Untreated Cells

Both rhGH and IGF-1 were added to untreated primary cortical cells in various concentrations ranging from 0.01 to 1000 nM for three consecutive days. At the end of the experiment, the mitochondrial function, as assessed by tetrazolium bromide (MTT) assay, was evaluated. There was no overall effect of either rhGH or IGF-1 treatment on untreated cells (ANOVA, *p* > 0.05) and no further *post hoc* analysis was carried out (Figure 1C,D). These results indicate that neither rhGH nor IGF-1 had any effects on the mitochondrial function in normal untreated cells.

### 2.3. rhGH, unlike IGF-1, Is Protective Against Methadone-Induced Membrane Damage 

**Figure 2:**
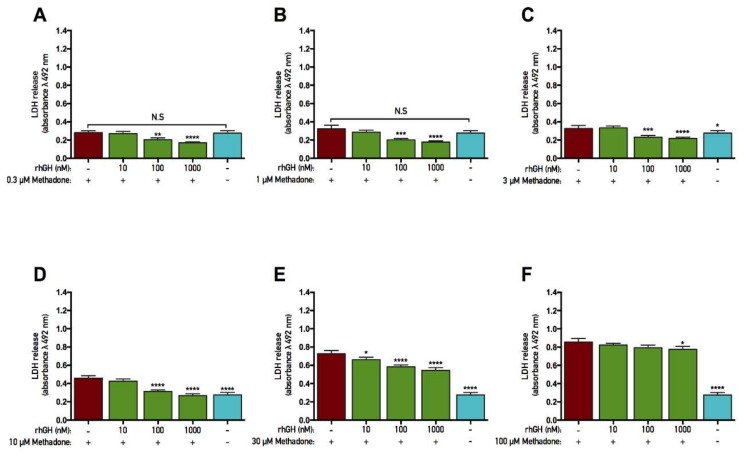
Recombinant human growth hormone protects against methadone-induced membrane damage. Membrane integrity was assessed by lactate dehydrogenase (LDH) assay. Recombinant human growth hormone (rhGH) was added with methadone for three consecutive days prior to analysis. (**A**) Effects of 0.3 µM methadone with or without 10–1000 nM rhGH. No significant difference was seen between methadone alone and control, but LDH levels were significantly lower when 0.3 µM methadone was co-treated with 100 and 1000 nM rhGH. (**B**) Effects of 1 µM methadone with or without 10–1000 nM rhGH. No significant difference was seen between methadone alone and control, but LDH levels were significantly lower when 1 µM methadone was co-treated with 100 and 1000 nM rhGH. (**C**) Effects of 3 µM methadone with or without 10–1000 nM rhGH. Significantly higher LDH release was observed when 3 µM methadone was given alone. The increased levels were significantly lower when 3 µM methadone was co-treated with 100 and 1000 nM rhGH. (**D**) Effects of 10 µM methadone with or without 10–1000 nM rhGH. Significantly higher LDH release was observed when 10 µM methadone was given alone. The increased levels were significantly lower when 10 µM methadone was co-treated with 100 and 1000 nM rhGH. (**E**) Effects of 30 µM methadone with or without 10–1000 nM rhGH. Significantly higher LDH release was observed when 30 µM methadone was given alone. The increased levels were significantly lower when 30 µM methadone were co-treated with 10, 100, and 1000 nM rhGH. (**F**) Effects of 100 µM methadone with or without 10–1000 nM rhGH. Significantly higher LDH release was observed when 100 µM methadone was given alone. The increased levels were significantly lower when 30 µM methadone was co-treated with 1000 nM rhGH. Statistics were calculated using two-way analysis of variance (ANOVA) and given a significant overall effect (*p* < 0.05), the data were further analyzed using Dunnet’s *post hoc* test and compared with results from cells treated with methadone alone. All data are presented as mean ± SEM in triplicate from five individual cultures. * *p* < 0.05, ** *p* < 0.01, *** *p* < 0.001, **** *p* < 0.0001.

**Figure 3:**
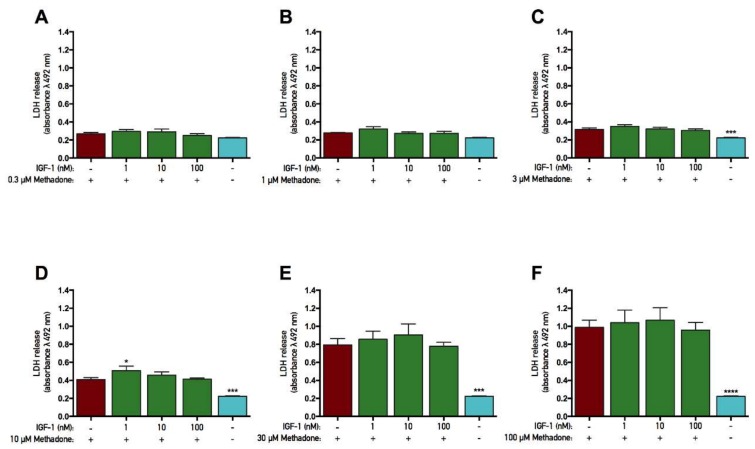
Insulin-like growth factor-1 does not protect against methadone-induced membrane damage. Membrane integrity was assessed by lactate dehydrogenase (LDH) assay. Insulin-like growth factor-1 (IGF-1) was added with methadone for three consecutive days prior to analysis. (**A**) Effects of 0.3 µM methadone with or without 1–100 nM IGF-1. No significant effects were observed. (**B**) Effects 1 µM methadone with or without 1–100 nM IGF-1. No significant effects were observed. (**C**) Effects 3 µM methadone with or without 1–100 nM IGF-1. Significantly higher LDH release was observed when 3 µM methadone was given alone. There was no significant difference in LDH release when 3 µM methadone was co-treated with IGF-1. (**D**) Effects 10 µM methadone with or without 1–100 nM IGF-1. Significantly higher LDH release was observed when 10 µM methadone was given alone. There was a significant increase of LDH release when 10 µM methadone was co-treated with 1 nM IGF-1, but no other differences were observed. (**E**) Effects 30 µM methadone with or without 1–100 nM IGF-1. Significantly higher LDH release was observed when 30 µM methadone was given alone. There was no significant difference when 30 µM methadone was co-treated with IGF-1. (**F**) Effects 100 µM methadone with or without 1–100 nM IGF-1. Significantly higher LDH release was observed when 100 µM methadone was given alone. There was no significant difference in LDH release when 100 µM methadone was co-treated with IGF-1. Statistics were calculated using two-way analysis of variance (ANOVA) and given a significant overall effect (*p* < 0.05), the data were further analyzed using Dunnet’s *post hoc* test and compared with results from cells treated with methadone alone. All data are presented as mean ± SEM in triplicate from five individual cultures. * *p* < 0.05,*** *p* < 0.001, **** *p* < 0.0001.

The opioid methadone was added to primary cortical cells in various concentrations ranging from 0.1 to 100 µM for three consecutive days. At the same time, each concentration of methadone was also co-treated with either rhGH (10–1000 nM) or IGF-1 (1–100 nM) prior to analysis of the membrane integrity (as assessed by LDH assay). There was an overall effect of treatment in all of the protection studies with both rhGH and IGF-1 on methadone-treated cells (as assessed by LDH assay, ANOVA *p* < 0.05). Further *post hoc* analysis revealed that 3, 10, 30, and 100 µM of methadone each induced significantly higher LDH release compared with control in both the rhGH and IGF-1 studies (Figure 2C–F and Figure 3C–F). No significant differences in LDH release were observed when cells were exposed to 0.1 or 1 µM methadone. These results demonstrate that three-day treatment with methadone causes significant damage to the cell membrane at concentrations starting from 3 µM. 

Furthermore, *post hoc* analysis revealed that both 100 and 1000 nM rhGH co-treated with 3, 10, or 30 µM methadone significantly decreased LDH release compared with each concentration of methadone alone (Figure 2C–E). Only 1000 nM rhGH was able to significantly decrease LDH release when co-treated with 100 µM methadone (Figure 2F). However, 10 nM rhGH significantly decreased LDH only when co-treated with 30 µM methadone compared with methadone alone (Figure 2E). The general membrane-stabilizing effects of rhGH treatment (as described in Section 2.2) were once again observed in the experiments using 0.3 and 1 µM methadone treatment, as these concentrations did not cause any significant differences in LDH release (Figure 2A,B).

There was no significant decrease in LDH release when IGF-1 was co-treated with either of the methadone doses (Figure 3A–F), although a significant increase in LDH was found when 1 nM IGF-1 was co-treated with 10 µM methadone compared with methadone alone (Figure 3D). These results indicate that rhGH, but not IGF-1, protects membrane integrity from methadone-induced damage, as lower levels of LDH correlate with its reduced leakage to cell media.

### 2.4. rhGH and IGF-1 Are Not Protective Against Methadone-Induced Mitochondrial Dysfunction

**Figure 4:**
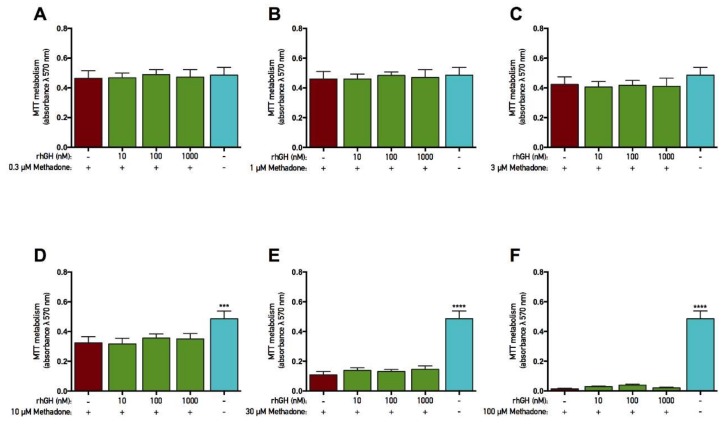
No protective effects of recombinant human growth hormone on mitochondrial function were found when co-treated with methadone. Mitochondrial function was assessed by tetrazolium bromide (MTT) assay. Recombinant human growth hormone (rhGH) was added with methadone for three consecutive days prior to analysis. (**A**) Effects of 0.3 µM with or without 10–1000 nM rhGH. There were no differences between groups. (**B**) Effects of 1 µM methadone with or without 10–1000 nM rhGH. There were no differences between groups. (**C**) Effects of 3 µM methadone with or without 10–1000 nM rhGH. There were no differences between groups. (**D**) Effects of 10 µM methadone with or without 10–1000 nM rhGH. A significant decrease in mitochondrial function was observed when 10 µM methadone was given alone. There was no significant difference in mitochondrial function when methadone was co-treated with rhGH. (**E**) Effects of 30 µM methadone with or without 10–1000 nM rhGH. A significant decrease in mitochondrial function was observed when 30 µM methadone was given alone. There was no significant difference in mitochondrial function when methadone was co-treated with rhGH. (**F**) Effects of 100 µM methadone with or without 10–1000 nM rhGH. A significant decrease in mitochondrial function was observed when 100 µM methadone was given alone. There was no significant difference in mitochondrial function when methadone was co-treated with rhGH. Statistics were calculated using two-way analysis of variance (ANOVA) and given a significant overall effect (*p* < 0.05), the data were further analyzed using Dunnet’s *post hoc* test and compared with results from cells treated with methadone alone. All data are presented as mean ± SEM in triplicate from five individual cultures. *** *p* < 0.001, **** *p* < 0.0001.

**Figure 5:**
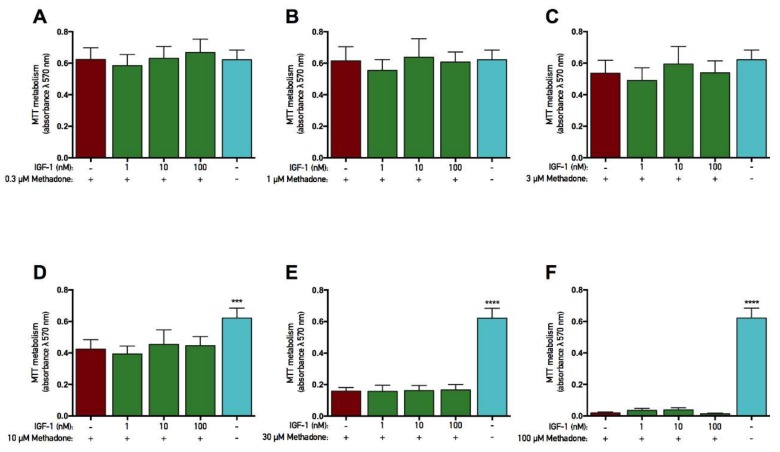
No protective effects of insulin-like growth factor-1 on mitochondrial function were found when co-treated with methadone. Mitochondrial function was assessed by tetrazolium bromide (MTT) assay. Insulin-like growth factor-1 (IGF-1) was added with methadone for three consecutive days prior to analysis. **(A)** Effects of 0.3 µM methadone with or without 1–100 nM IGF-1. There were no differences between groups. **(B)** Effects of 1 µM methadone with or without 1–100 nM IGF-1. There were no differences between groups. **(C)** Effects of 3 µM methadone with or without 1–100 nM IGF-1. No significant effects were observed. **(D)** Effects of 10 µM methadone with or without 1–100 nM IGF-1. A significant decrease in mitochondrial function was observed when 10 µM methadone was given alone. There was no significant difference in mitochondrial function when methadone was co-treated with IGF-1. **(E)** Effects of 30 µM methadone with or without 1–100 nM IGF-1. A significant decrease in mitochondrial function was observed when 30 µM methadone was given alone. There was no significant difference in mitochondrial function when methadone was co-treated with IGF-1. **(F)** Effects of 100 µM methadone with or without 1–100 nM IGF-1. A significant decrease in mitochondrial function was observed when 100 µM methadone was given alone. There was no significant difference in mitochondrial function when methadone was co-treated with IGF-1. Statistics were calculated using two-way analysis of variance (ANOVA) and given a significant overall effect (*p* < 0.05), the data were further analyzed using Dunnet’s *post hoc* test and compared with results from cells treated with methadone alone. All data are presented as mean ± SEM in triplicate from three individual cultures. *** *p* < 0.001, **** *p* < 0.0001.

The opioid methadone was added to primary cortical cells in various concentrations ranging from 0.1 to 100 µM for three consecutive days. At the same time, each concentration was also co-treated with either rhGH (10–1000 nM) or IGF-1 (1–100 nM) prior to analysis of the mitochondrial function (as assessed by MTT assay). In both rhGH and IGF-1 protection studies, there was an overall effect of treatment in the experiments with 10, 30, and 100 µM methadone (ANOVA, *p* < 0.05). Further *post hoc* analysis revealed that 10, 30, and 100 µM methadone each significantly decreased MTT metabolism compared with control (Figure 4D–F and Figure 5D–F). These results demonstrate that three-day treatment with methadone reduces mitochondrial activity in concentrations starting from 10 µM. However, there was no significant difference when rhGH or IGF-1 was co-treated with methadone compared with methadone alone, although a small positive numerical difference was found with rhGH treatment (Figure 4 and Figure 5, respectively). For instance, when methadone was co-treated with 1000 nM rhGH, 6% and 7% higher mitochondrial activity was observed using 10 and 30 µM methadone, respectively. There was also an overall effect of treatment in the IGF-1 protection study with 3 µM methadone (ANOVA, *p* = 0.0225), but *post hoc* analysis revealed no significant differences in MTT metabolism. 

### 2.5. rhGH and IGF-1 Restore Mitochondria Function Following Acute Methadone Treatment

**Figure 6:**
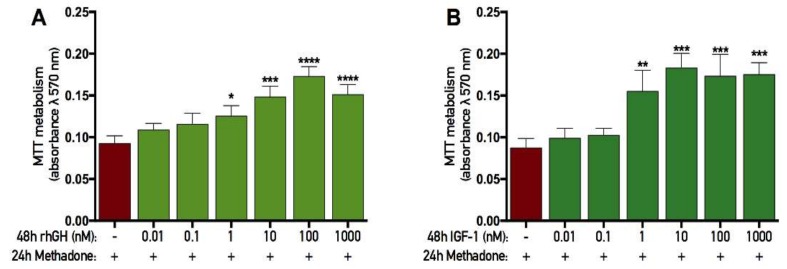
Recovery effects of recombinant human growth hormone and insulin-like growth factor-1 on mitochondrial function following methadone-induced toxicity. Mitochondrial function was assessed by tetrazolium bromide (MTT) assay. Methadone (60 µM) was added for 24 h and then washed out. The following 48 h, recombinant human growth hormone (rhGH) and insulin-like growth factor-1 (IGF-1) were added for two consecutive days prior to analysis. (**A**) The 48 h recovery effect of rhGH in cells with methadone-induced mitochondrial dysfunction. A significant increase in mitochondrial function was observed using 1, 10, 100, and 1000 nM rhGH. (**B**) The 48 h recovery effect of IGF-1 in cells with methadone-induced mitochondrial dysfunction. A significant increase in mitochondrial function was observed using 1, 10, 100, and 1000 nM IGF-1. Statistics were calculated using two-way analysis of variance (ANOVA) and given a significant overall effect (*p* < 0.05), the data were further analyzed using Dunnet’s *post hoc* test compared to cells exposed to control during the 48 h recovery phase. All data are presented as mean ± SEM in triplicate from five individual cultures. * *p* < 0.05, ** *p* < 0.01, *** *p* < 0.001, **** *p* < 0.0001.

A single treatment of 60 µM methadone was added to primary cortical cells for 24 h. After this period, the cells were washed and subsequently treated with either rhGH or IGF-1 for 48 h. At the end of each experiment, the mitochondrial function (as assessed by MTT assay) was evaluated. There was an overall effect of treatment in the recovery experiment with rhGH (ANOVA, *p* < 0.0001), and further *post hoc* analysis revealed that 48 h treatment with 1, 10, 100, and 1000 nM rhGH significantly increased MTT metabolism compared with cells treated with control during the recovery period (Figure 6A). A more detailed analysis revealed that when cells were treated with control for 48 h after the initial 24 h methadone treatment, the mitochondrial activity was calculated to be 24% (in % of untreated control cells). As the control treatment consisted of water vehicle, it is safe to say that methadone caused these negative effects. However, when rhGH was added in the 48h recovery period, the mitochondrial activity increased to 31%, 36%, 43%, and 38% using 1, 10, 100, and 1000 nM rhGH, respectively. 

Similar to the rhGH results, there was an overall effect of treatment in the IGF-1 experiment (as assessed by MTT assay; ANOVA, *p* < 0.0001), and further *post hoc* analysis revealed that 48 h treatment of 1, 10, 100, and 1000 nM IGF-1 significantly increased MTT metabolism compared with cells treated with control during the recovery period (Figure 6B). A more detailed analysis revealed that when cells were treated with control for 48 h after the initial 24 h methadone treatment, the mitochondrial activity was calculated to be 23% of untreated cells. However, when IGF-1 was added in the 48 h recovery period, the mitochondrial activity increased to 45%, 49%, 45%, and 45% using 1, 10, 100, and 1000 nM, respectively. These results, with a maximum 19% increase using 100 nM rhGH and a maximum 26% increase using 10 nM IGF-1, indicate that an ongoing restorative effect on mitochondrial activity is present when injured cells are treated with rhGH and IGF-1. 

### 2.6. rhGH and IGF-1 Restore Membrane Integrity Following Acute Methadone Treatment

**Figure 7:**
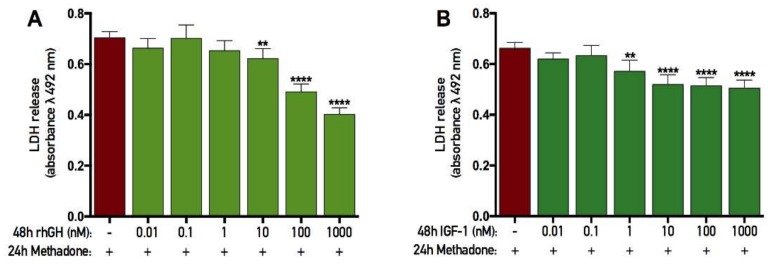
Recovery effects of recombinant human growth hormone and insulin-like growth factor-1 on membrane integrity following methadone-induced toxicity. Membrane integrity was assessed by lactate dehydrogenase (LDH) assay. Methadone (60 µM) was added for 24 h and then washed out. The following 48 h, recombinant human growth hormone (rhGH) and insulin-like growth factor-1 (IGF-1) were added for two consecutive days prior to analysis. (**A**) The 48 h recovery effect of rhGH in cells with methadone-induced membrane damage. A significant reduction in LDH release during the recovery phase was observed using 10, 100, and 1000 nM rhGH. (**B**) The 48 h recovery effect of IGF-1 in cells with methadone-induced membrane damage. A significant reduction in LDH release during the recovery phase was observed using 1, 10, 100, and 1000 nM IGF-1. Statistics were calculated using two-way analysis of variance (ANOVA) and given a significant overall effect (*p* < 0.05), the data were further analyzed using Dunnet’s *post hoc* test compared to cells exposed to control during the 48 h recovery phase. All data are presented as mean ± SEM in triplicate from five individual cultures. ** *p* < 0.01, **** *p* < 0.0001.

A single treatment of 60 µM methadone was added to primary cortical cells for 24 h. After this period, the cells were washed and subsequently treated with either rhGH or IGF-1 for 48 h. At the end of each experiment, the membrane integrity (as assessed by LDH assay) was evaluated. There was an overall effect of treatment in the recovery experiment with rhGH (ANOVA, *p* < 0.0001), and further *post hoc* analysis revealed that 48 h treatment with 10, 100, and 1000 nM rhGH significantly reduced LDH in cell media compared with cells treated with control during the recovery period (Figure 7A). As the cells were washed after initial treatment with methadone, the data from this assay only display the release of LDH during the 48 h recovery period. A more detailed analysis revealed that when cells were treated with control for 48 h after the initial 24 h methadone treatment, the release of LDH was calculated to be 48% of maximum release. However, when rhGH was added for 48 h, LDH release was calculated to be 42%, 33%, and 27% using 10, 100, and 1000 nM rhGH, respectively.

In the IGF-1 experiment, there was an overall effect of treatment (as assessed by LDH assay; ANOVA, *p* < 0.0001), and further *post hoc* analysis revealed that 48 h treatment of 1, 10, 100, and 1000 nM IGF-1 significantly reduced LDH in cell media compared with cells treated with control during the recovery period (Figure 7B). A more detailed analysis revealed that when cells were treated with control for 48 h after the initial 24 h methadone treatment, the release of LDH was calculated to be 45% of maximum release. However, when IGF-1 was added for 48 h, LDH release was calculated to be 39%, 35%, 35%, and 34% using 1, 10, 100, and 1000 nM IGF-1, respectively. These results, with a maximum of 21% decrease in LDH release using 1000 nM rhGH and a maximum of 11% decrease using 1000 nM IGF-1, indicate that both rhGH and IGF-1 effectively enhance membrane integrity following injury.

## 3. Discussion

The main findings from the present study demonstrate that rhGH, but not IGF-1, protects cells from methadone-induced cell damage and that both rhGH and IGF-1 have restorative effects when cells are pretreated with methadone. These results confirm the role of GH in protecting cells from various insults [15,16,17] and further support previous studies that present GH as an effective rehabilitating treatment against various CNS injuries [12,13,44]. 

We previously reported that acute 24 h treatment with rhGH protects mitochondrial function and stabilizes membrane integrity in primary cortical cells exposed to acute 24 h treatment with methadone [18]. In the present study, we assessed the protective effect of rhGH following a three-day treatment paradigm in order to mimic repeated administration and were able to replicate its beneficial effects in protecting membrane integrity (as assessed by LDH assay), but no effect was seen on mitochondrial function (as assessed by MTT assay). All concentrations of rhGH demonstrated membrane-stabilizing effects, with the most effective doses able to reduce LDH back to control levels. With certain doses, although significant, the numerical decrease was quite small, which may limit the biological relevance. The reason why rhGH is more effective in protecting membrane integrity than mitochondrial activity remains elusive, but it may depend on the signaling pathway initiated by methadone. Methadone has been suggested to induce cell death through both a caspase 3/7–dependent apoptotic pathway [18] and a necrotic-like pathway by depleting cellular adenosine triphosphate (ATP) levels [38]. However, it is likely that these two signaling events occur at the same time. Compared to the MTT assay, the LDH assay is more likely to be associated with necrotic-like cell death, which generally involves swelling of the cytosol prior to membrane loss (and leakage of LDH into the cell medium). As rhGH has been reported to have no effect in reducing methadone-induced caspase 3/7 activity in primary cortical cells [18], it is tempting to say that GH may be more efficient in protecting cells against necrotic-like cell damage. However, this may also depend on the cell type and its origin, as rhGH has been reported to reduce caspase 3/7 activity in hippocampal cell cultures [19]. Another interesting result from the present study is that when rhGH was given alone, a small membrane-stabilizing effect was observed compared to untreated cells. Similar results have been described previously [18] and may suggest that GH alone increases the general health of primary cortical cell cultures. For instance, rhGH may effectively counteract spontaneous cell death when cultured cells are grown in an artificial environment. However, this was significantly decreased only when the highest dose of rhGH was used, thus the biological relevance of this data may be limited. 

Interestingly, no protective effects of co-treatment with methadone and IGF-1 were observed using either of the assays. However, there was a clear restorative effect of IGF-1 in cells damaged by methadone. Similar to the IGF-1 results, rhGH also effectively potentiated both membrane integrity and mitochondrial function in methadone-damaged cells. There was a clear numerical difference between the restorative effects of rhGH and IGF-1, in that IGF-1 treatment was more effective in increasing mitochondrial activity, while rhGH was more effective in recovering membrane integrity when given during the 48 h recovery period. In fact, rhGH was able to reduce LDH levels back to control levels during the recovery period, further confirming the results from the protection study. These findings are in agreement with the positive rehabilitating effects of GH, as seen in the treatment of patients suffering from various brain traumas [12,13,44], which may be linked to the strong effects both hormones have on neurogenesis and gliogenesis [8,9]. Taken together with the protection results, these data indicate that GH and IGF-1 have different roles in the CNS. This may explain why GH-treated cells display a more typical dose response compared to IGF-1 treated cells. It is well known that IGF-1 mediates the effects of GH in the periphery, but less is known about the action of these substances in the brain. As GH and the somatotrophic axis stimulate the peripheral release of IGF-1 from the liver, it is possible that a similar regulation process occurs locally in the brain. The effect of IGF-1 may also differ depending on whether it is released by a local autocrine/paracrine mechanism or enters the brain via the blood–brain barrier [45]. 

Both the protective effects of rhGH and the beneficial recovery effects of rhGH and IGF-1 support various studies associated with cognition. Several studies address GH as a cognitive enhancer that displays positive effects in various models, with a focus on cognition [4,7,10,11]. We, and others, have examined the positive effects of GH in restoring or enhancing cognitive capabilities in models using substance-induced cognitive dysfunction, including opioids, with promising results [5,6,14]. However, the exact mechanism of how these hormones exert their effects is not fully known. Several studies suggest that the mechanism behind these positive effects is associated with the *N*-methyl-d-aspartate (NMDA) receptor [2,4,7,18,46], which is known to promote synaptic strength, long-term potentiation, and memory and learning [47]. Therefore, GH or IGF-1 may be considered as a suitable agent for the treatment of damage induced by drugs or diseases that target the NMDA receptor and disrupt cognitive function. The opioid methadone, also known for its noncompetitive inhibitory effects on the NMDA receptor [48,49], is one such drug. Hence, the positive effects seen from rhGH (and possibly IGF-1) treatment may be related to the NMDA receptor. This has been demonstrated previously, with rhGH treatment effectively normalizing high methadone-induced gene expression of NMDA receptor subunits [18].

There are, however, limitations to the present study. For instance, the use of high doses of both methadone and rhGH/IGF-1 may deviate from physiologic concentrations in vivo. However, the high doses of methadone enabled us to detect small changes in cell viability, and we hypothesize that the same results seen in this study also occur in vivo, albeit at different concentrations. The present study was not designed to mimic an in vivo study, but rather to investigate the beneficial properties of GH and IGF-1 treatment. There may also be confounding data regarding the use of rhGH instead of rat GH in our cultures due to selectivity differences, with hGH also affecting the prolactin receptors [50].

In conclusion, in the present study we have demonstrated that rhGH, but not IGF-1, protects primary cortical cell cultures against methadone-induced cell damage and that both rhGH and IGF-1 effectively potentiate the recovery of cells treated with methadone. This study further highlights the beneficial effects of GH and IGF-1 in the CNS, which also supports the hypothesis that these hormones act as neuroprotectants and possibly also as effective cognitive enhancers. Furthermore, our results indicate that GH and IGF-1 may have different roles depending on whether they are involved in neuroprotection or during neurorepair. The present study also provides further insight into how GH or IGF-1 can be used as a possible agent for the treatment of cognitive disabilities or for neuronal recovery in patients suffering from various kinds of brain damage. Many of these studies highlight that the beneficial effects from GH are associated with NMDA receptor transmission, which may also play a crucial role in protecting or restoring damaged cells. However, the exact molecular mechanism (or mechanisms) mediating these effects remains unknown and requires further study. 

## 4. Materials and Methods

All animal experiments were approved by the Uppsala animal ethics committee (number C14/15, date 2 August 2014) according to the Swedish guidelines regarding animal experiments (Animal Welfare Act SFS1998:56) and the European Communities directive (86/609/EEC).

### 4.1. Primary Cell Cultures

Mixed glial and neuronal primary cortical cell cultures were harvested from embryonic day 17 (E17) Wistar rats (Charles River, Sulzfeld, Germany) and prepared as described previously [18,51]. Briefly, the brains from E17 Wistar rats were removed and cortices were carefully dissected and placed in Hank’s Balanced Salt solution (Thermo Fisher Scientific, Waltham, MA, USA). The tissues were enzymatically digested using 0.2 mg/mL trypsin (Sigma-Aldrich, St. Louis, MO, USA) at 37 °C for 10 min, and the reaction was terminated using 0.52 mg/mL trypsin inhibitor (Sigma Aldrich). The digested tissue was mechanically dissociated using a 1 mL pipette and the cell suspension was centrifuged for 5 min at 3200 rpm. The acquired cell pellet was resuspended in neurobasal medium (NBM; Thermo Fisher Scientific) supplemented with 10% (*v/v*) fetal bovine serum (Thermo Fisher Scientific), 2% (*v/v*) B27 (Thermo Fisher Scientific), 0.5 mM GlutaMAX™ (Thermo Fischer Scientific), and 100 U/mL penicillin/streptomycin (Thermo Fisher Scientific). Cells were counted using the Countess™ II Automated Cell Counter (Thermo Fisher Scientific) prior to seeding them into a 96-well tissue culture plate precoated with 50 µg/mL poly-D-lysine (Sigma-Aldrich) at a density of 1 × 10^5^ cells/well. Cells were placed in a humidified incubator at 37 °C, 5% CO_2_, and replaced with fresh serum-free media containing an additional 2% (*v/v*) B27 the next day. Cells were monitored daily and a half media change was performed at 3 days in vitro (DIV). Drug treatments were initiated at 7 DIV, when the cells had grown a considerable network of dendrites. As cells were maintained in serum-free NBM, which favors neuronal growth; our mixed primary cortical cultures are known to contain more neurons than glial cells, with an approximate ratio of 60/40. The results obtained from the present study highlight the effects on mixed cortical cells and do not discriminate between different cell types.

### 4.2. Treatments

#### 4.2.1. GH and IGF-1 Treatment 

In order to examine the general effects of recombinant human GH (rhGH) and IGF-1, a 3 day repeated experiment was carried out. At 7 DIV, 0.01–1000 nM rhGH (Genotropin^®^, Pfizer, Sweden) or IGF-1 (Sigma-Aldrich) was administered to the cells in triplicate for 3 days, with each treatment given once per day. For each separate experiment, a negative control containing cell media and water was added using the same treatment regime as stated above.

#### 4.2.2. GH/IGF-1 and Protection Experiments

To assess the role of rhGH and IGF-1 in protecting primary cortical cells exposed to methadone, a 3 day repeated experiment was carried out. At 7 DIV, methadone hydrochloride (Sigma-Aldrich) was added to cortical cells ranging from 0.3 to 100 µM in triplicate. At the same time, rhGH (10, 100, and 1000 nM) or IGF-1 (1, 10, and 100 nM) was added with methadone in another triplicate set. Each concentration of methadone was examined with or without each concentration of rhGH or IGF-1, and the effects were evaluated at the end of the 3 day incubation period. For each separate experiment, a negative control containing cell media and water was added using the same treatment regime as stated above.

#### 4.2.3. GH/IGF-1 and Recovery Experiments

To assess the restorative role of rhGH and IGF-1 on primary cortical cells following acute methadone exposure, a 3 day repeated experiment was carried out. At 7 DIV, methadone (60 µM) was added in triplicate to cortical cells for 24 h and then removed and replaced with fresh media containing rhGH (10, 100, and 1000 nM) or IGF-1 (1, 10, and 100 nM). Both GH and IGF-1 were added for 48 h after methadone exposure, each dose given in 24 h intervals. One triplicate set of control wells containing methadone for 24 h with water added for the next 48 h was also included. To ensure normal and healthy growth of cells, a triplicate set of control wells without any methadone or GH/IGF-1 added was used throughout the experiment and equal media changes were carried out as stated above. These control wells were not included in the statistical analysis.

### 4.3. Mitochondrial Function

Tetrazolium bromide salt (MTT) is metabolized to the insoluble purple product formazan in active mitochondria and can thus be used as a marker for mitochondrial function. At the end of each experiment, MTT (Sigma-Aldrich) was added at a final concentration of 1 mg/mL and placed in a humidified incubator (37 °C, 5% (*v/v*) CO_2_) for 30 min prior to lysing the cells with 100% (*v/v*) dimethyl sulfoxide. The plates were kept in the dark for 10 min prior to analysis of absorbance using a plate-reader (FLUOstar Omega, Ortenberg, Germany) at 570 nM. The MTT assay was carried out in both the protection and recovery experiments.

### 4.4. Lactate Dehydrogenase

Lactate dehydrogenase (LDH) is located in the cytoplasm and leaks into the cell media upon damage to the cell membrane. The LDH assay thus acts as a marker of membrane integrity or damage. At the end of each experiment, the media was transferred from the treated tissue culture plate to a new empty plate prior to analysis. An LDH detection kit (Sigma-Aldrich) was used according to the manufacturer’s instructions. Briefly, the kit detects LDH in cell media by utilizing an enzymatic reaction that produces a dark red formazan product that corresponds to available LDH present in the media. In order to assess membrane integrity, the absorbance of the red formazan product was quantified using a plate-reader at 492 nM. The LDH assay was carried out in both the protection and recovery experiments. However, as a full media change was performed after the acute 24 h methadone treatment in the recovery studies, the LDH assay only measured the released LDH during the recovery period in these experiments.

### 4.5. Statistical Analysis

Statistical analysis was performed on raw data using GraphPad Prism (version 6.0 h). Primary cortical cells harvested from one individual rat were considered as one culture (*n* = 1), and each culture received all treatments. The data from each experiment were analyzed using a randomized block analysis of variance (ANOVA) with treatment and experiment (culture ID) as factors to account for differences between cultures [52]. Given a significant overall effect of treatment, further analysis was carried out using Dunnet’s *post hoc* test. The differences were considered statistically significant at *p* < 0.05. Data are expressed as standard error of the mean (±SEM), with data gathered from a minimum of three individual cultures.

## Abbreviations

ANOVA	Analysis of variance
CNS	Central nervous system
DIV	Day(s) in vitro
E17	Embryonic day 17
GH	Growth hormone
IGF-1	Insulin-like growth factor-1
LDH	Lactate dehydrogenase
LTP	Long-term potentiation
MTT	Tetrazolium bromide
NBM	Neurobasal medium
NMDA	*N*-methyl-d-aspartate
rhGH	Recombinant human growth hormone
SEM	Standard error of the mean

## References

[1] Nyberg F. (2000). Growth hormone in the brain: Characteristics of specific brain targets for the hormone and their functional significance. Front. Neuroendocrinol..

[2] Nyberg F., Hallberg M. (2013). Growth hormone and cognitive function. Nat. Rev. Endocrinol..

[3] Le Greves M., Zhou Q., Berg M., Le Greves P., Fholenhag K., Meyerson B., Nyberg F. (2006). Growth hormone replacement in hypophysectomized rats affects spatial performance and hippocampal levels of NMDA receptor subunit and PSD-95 gene transcript levels. Exp. Brain Res..

[4] Ramis M., Sarubbo F., Sola J., Aparicio S., Garau C., Miralles A., Esteban S. (2013). Cognitive improvement by acute growth hormone is mediated by NMDA and AMPA receptors and MEK pathway. Prog. Neuro.-Psychoph..

[5] Gronbladh A., Johansson J., Nostl A., Nyberg F., Hallberg M. (2013). GH improves spatial memory and reverses certain anabolic androgenic steroid-induced effects in intact rats. J. Endocrinol..

[6] Enhamre-Brolin E., Carlsson A., Hallberg M., Nyberg F. (2013). Growth hormone reverses streptozotocin-induced cognitive impairments in male mice. Behav. Brain Res..

[7] Studzinski A.L., Barros D.M., Marins L.F. (2015). Growth hormone (GH) increases cognition and expression of ionotropic glutamate receptors (AMPA and NMDA) in transgenic zebrafish (Danio rerio). Behav. Brain Res..

[8] Heredia M., Fuente A., Criado J., Yajeya J., Devesa J., Riolobos A.S. (2013). Early growth hormone (GH) treatment promotes relevant motor functional improvement after severe frontal cortex lesion in adult rats. Behav. Brain Res..

[9] Aberg D. (2010). Role of the growth hormone/insulin-like growth factor 1 axis in neurogenesis. Endocr. Dev..

[10] Falleti M.G., Maruff P., Burman P., Harris A. (2006). The effects of growth hormone (GH) deficiency and GH replacement on cognitive performance in adults: A meta-analysis of the current literature. Psychoneuroendocrinology.

[11] Elbornsson M., Horvath A., Gotherstrom G., Bengtsson B.A., Johannsson G., Svensson J. (2017). Seven years of growth hormone (GH) replacement improves quality of life in hypopituitary patients with adult-onset GH deficiency. Eur. J. Endocrinol..

[12] Devesa J., Reimunde P., Devesa P., Barbera M., Arce V. (2013). Growth hormone (GH) and brain trauma. Horm. Behav..

[13] Devesa J., Lema H., Zas E., Munin B., Taboada P., Devesa P. (2016). Learning and Memory Recoveries in a Young Girl Treated with Growth Hormone and Neurorehabilitation. J. Clin. Med..

[14] Rhodin A., von Ehren M., Skottheim B., Gronbladh A., Ortiz-Nieto F., Raininko R., Gordh T., Nyberg F. (2014). Recombinant human growth hormone improves cognitive capacity in a pain patient exposed to chronic opioids. Acta Anaesthesiol. Scand..

[15] Scheepens A., Sirimanne E.S., Breier B.H., Clark R.G., Gluckman P.D., Williams C.E. (2001). Growth hormone as a neuronal rescue factor during recovery from CNS injury. Neuroscience.

[16] Alba-Betancourt C., Luna-Acosta J.L., Ramirez-Martinez C.E., Avila-Gonzalez D., Granados-Avalos E., Carranza M., Martinez-Coria H., Aramburo C., Luna M. (2013). Neuro-protective effects of growth hormone (GH) after hypoxia-ischemia injury in embryonic chicken cerebellum. Gen. Comp. Endocrinol..

[17] Martinez-Moreno C.G., Fleming T., Carranza M., Avila-Mendoza J., Luna M., Harvey S., Aramburo C. (2018). Growth hormone protects against kainate excitotoxicity and induces BDNF and NT3 expression in chicken neuroretinal cells. Exp. Eye Res..

[18] Nylander E., Gronbladh A., Zelleroth S., Diwakarla S., Nyberg F., Hallberg M. (2016). Growth hormone is protective against acute methadone-induced toxicity by modulating the NMDA receptor complex. Neuroscience.

[19] Svensson A.L., Bucht N., Hallberg M., Nyberg F. (2008). Reversal of opiate-induced apoptosis by human recombinant growth hormone in murine foetus primary hippocampal neuronal cell cultures. Proc. Natl. Acad. Sci. USA.

[20] Pan W., Yu Y., Cain C.M., Nyberg F., Couraud P.O., Kastin A.J. (2005). Permeation of growth hormone across the blood-brain barrier. Endocrinology.

[21] Lai Z.N., Emtner M., Roos P., Nyberg F. (1991). Characterization of putative growth hormone receptors in human choroid plexus. Brain Res..

[22] Le Roith D., Bondy C., Yakar S., Liu J.L., Butler A. (2001). The somatomedin hypothesis: 2001. Endocr. Rev..

[23] Schneider H.J., Pagotto U., Stalla G.K. (2003). Central effects of the somatotropic system. Eur. J. Endocrinol..

[24] Bluthe R.M., Frenois F., Kelley K.W., Dantzer R. (2005). Pentoxifylline and insulin-like growth factor-I (IGF-I) abrogate kainic acid-induced cognitive impairment in mice. J. Neuroimmunol..

[25] D'Mello S.R., Galli C., Ciotti T., Calissano P. (1993). Induction of apoptosis in cerebellar granule neurons by low potassium: Inhibition of death by insulin-like growth factor I and cAMP. Proc. Natl. Acad. Sci. USA.

[26] Parrizas M., Saltiel A.R., LeRoith D. (1997). Insulin-like growth factor 1 inhibits apoptosis using the phosphatidylinositol 3′-kinase and mitogen-activated protein kinase pathways. J. Biol. Chem..

[27] Johnston B.M., Mallard E.C., Williams C.E., Gluckman P.D. (1996). Insulin-like growth factor-1 is a potent neuronal rescue agent after hypoxic-ischemic injury in fetal lambs. J. Clin. Invest..

[28] Beck K.D., Powell-Braxton L., Widmer H.R., Valverde J., Hefti F. (1995). Igf1 gene disruption results in reduced brain size, CNS hypomyelination, and loss of hippocampal granule and striatal parvalbumin-containing neurons. Neuron.

[29] Sonntag W.E., Ramsey M., Carter C.S. (2005). Growth hormone and insulin-like growth factor-1 (IGF-1) and their influence on cognitive aging. Ageing Res. Rev..

[30] van Dam P.S., Aleman A. (2004). Insulin-like growth factor-I, cognition and brain aging. Eur. J. Pharmacol..

[31] Andersen J.M., Olaussen C.F., Ripe A., Morland J. (2011). Long-term methadone treatment impairs novelty preference in rats both when present and absent in brain tissue. Pharmacol. Biochem. Be..

[32] Hepner I.J., Homewood J., Taylor A.J. (2002). Methadone disrupts performance on the working memory version of the Morris water task. Physiol. Behav..

[33] Cummins E., Allen C.P., Ricchetti A., Boughner E., Christenson K., Haines M., Limebeer C.L., Parker L.A., Leri F. (2012). The effects of acute and chronic steady state methadone on memory retrieval in rats. Psychopharmacology.

[34] Tramullas M., Martinez-Cue C., Hurle M.A. (2007). Chronic methadone treatment and repeated withdrawal impair cognition and increase the expression of apoptosis-related proteins in mouse brain. Psychopharmacology.

[35] Schiltenwolf M., Akbar M., Hug A., Pfuller U., Gantz S., Neubauer E., Flor H., Wang H.L. (2014). Evidence of Specific Cognitive Deficits in Patients with Chronic Low Back Pain under Long-Term Substitution Treatment of Opioids. Pain Physician.

[36] Zeng H., Su D.Q., Jiang X., Zhu L., Ye H.S. (2016). The similarities and differences in impulsivity and cognitive ability among ketamine, methadone, and non-drug users. Psychiat. Res..

[37] Konijnenberg C., Melinder A. (2011). Prenatal exposure to methadone and buprenorphine: A review of the potential effects on cognitive development. Child Neuropsychol..

[38] Perez-Alvarez S., Cuenca-Lopez M.D., de Mera R.M., Puerta E., Karachitos A., Bednarczyk P., Kmita H., Aguirre N., Galindo M.F., Jordan J. (2010). Methadone induces necrotic-like cell death in SH-SY5Y cells by an impairment of mitochondrial ATP synthesis. Biochim. Biophys. Acta.

[39] Mao J., Sung B., Ji R.R., Lim G. (2002). Neuronal apoptosis associated with morphine tolerance: Evidence for an opioid-induced neurotoxic mechanism. J. Neurosci..

[40] Eisch A.J., Barrot M., Schad C.A., Self D.W., Nestler E.J. (2000). Opiates inhibit neurogenesis in the adult rat hippocampus. Proc. Natl. Acad. Sci. USA.

[41] Younger J.W., Chu L.F., D’Arcy N.T., Trott K.E., Jastrzab L.E., Mackey S.C. (2011). Prescription opioid analgesics rapidly change the human brain. Pain.

[42] Mahic M., Fredheim O.M., Borchgrevink P.C., Skurtveit S. (2015). Use of prescribed opioids by children and adolescents: Differences between Denmark, Norway and Sweden. Eur. J. Pain.

[43] Han B., Compton W.M., Jones C.M., Cai R. (2015). Nonmedical Prescription Opioid Use and Use Disorders Among Adults Aged 18 Through 64 Years in the United States, 2003–2013. JAMA.

[44] Ong L.K., Chow W.Z., TeBay C., Kluge M., Pietrogrande G., Zalewska K., Crock P., Aberg N.D., Bivard A., Johnson S.J. (2018). Growth Hormone Improves Cognitive Function After Experimental Stroke. Stroke.

[45] Torres-Aleman I. (2010). Toward a comprehensive neurobiology of IGF-I. Dev. Neurobiol..

[46] Le Greves M., Steensland P., Le Greves P., Nyberg F. (2002). Growth hormone induces age-dependent alteration in the expression of hippocampal growth hormone receptor and *N*-methyl-d-aspartate receptor subunits gene transcripts in male rats. Proc. Natl. Acad. Sci. USA.

[47] Tsien J.Z., Huerta P.T., Tonegawa S. (1996). The essential role of hippocampal CA1 NMDA receptor-dependent synaptic plasticity in spatial memory. Cell.

[48] Ebert B., Andersen S., Krogsgaard-Larsen P. (1995). Ketobemidone, methadone and pethidine are non-competitive *N*-methyl-d-aspartate (NMDA) antagonists in the rat cortex and spinal cord. Neurosci. Lett..

[49] Callahan R.J., Au J.D., Paul M., Liu C., Yost C.S. (2004). Functional inhibition by methadone of *N*-methyl-d-aspartate receptors expressed in Xenopus oocytes: Stereospecific and subunit effects. Anesth. Analg..

[50] Bartke A., Kopchick J.J. (2015). The forgotten lactogenic activity of growth hormone: Important implications for rodent studies. Endocrinology.

[51] Diwakarla S., Nylander E., Gronbladh A., Vanga S.R., Khan Y.S., Gutierrez-de-Teran H., Ng L., Pham V., Savmarker J., Lundback T. (2016). Binding to and Inhibition of Insulin-Regulated Aminopeptidase by Macrocyclic Disulfides Enhances Spine Density. Mol. Pharmacol..

[52] Lew M. (2007). Good statistical practice in pharmacology. Problem 2. Br. J. Pharmacol..

